# Dichlorido[*N*-(2-pyridylmethyl­idene)benzene-1,4-diamine]zinc(II)

**DOI:** 10.1107/S1600536810015138

**Published:** 2010-04-30

**Authors:** Yun-Fen Shi, Qiao-Hua Feng, Wen-Jie Zhao, Yun-Bo Shi, Peng Zhan

**Affiliations:** a School of Chemical Engineering, Northeast Dianli University, Jilin 132012, People’s Republic of China; b The Higher Educational Key Laboratory for Measuring Control Technology and Instrumentations of Heilongjiang Province, Measurement Control Tech Communications Engineering College, Harbin University of Science and Technology, Harbin 150080, People’s Republic of China; c School of Materials Science and Engineering, East China University of Science and Technology, Shanghai 200237, People’s Republic of China

## Abstract

In the title compound, [ZnCl_2_(C_12_H_11_N_3_)], the Zn^II^ atom is four-coordinated by two N atoms from an *N*-(2-pyridylmethyl­ene)benzene-1,4-diamine ligand and two Cl atoms in a distorted tetra­hedral geometry. In the crystal, the complex mol­ecules are connected by N—H⋯Cl and C—H⋯Cl hydrogen bonds into a two-dimensional layer structure parallel to (110).

## Related literature

For general background to zinc(II) complexes with Schiff base ligands, see: Su *et al.* (1999 ▶); Ye *et al.* (2005 ▶).
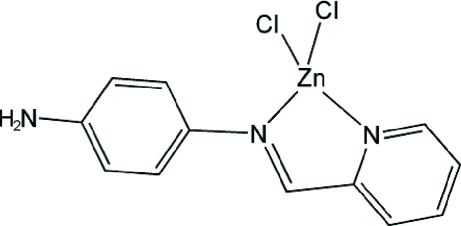


## Experimental

### 

#### Crystal data

[ZnCl_2_(C_12_H_11_N_3_)]
*M* *_r_* = 333.53
Triclinic, 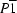

*a* = 7.5004 (15) Å
*b* = 9.1168 (18) Å
*c* = 10.186 (2) Å
α = 84.36 (3)°
β = 82.27 (3)°
γ = 74.19 (3)°
*V* = 662.7 (3) Å^3^
*Z* = 2
Mo *K*α radiation
μ = 2.24 mm^−1^
*T* = 293 K
0.20 × 0.18 × 0.16 mm

#### Data collection

Rigaku R-AXIS RAPID diffractometer
Absorption correction: multi-scan (*ABSCOR*; Higashi, 1995 ▶) *T*_min_ = 0.645, *T*_max_ = 0.699
6557 measured reflections
3000 independent reflections
2345 reflections with *I* > 2σ(*I*)
*R*_int_ = 0.027

#### Refinement

*R*[*F* ^2^ > 2σ(*F* ^2^)] = 0.038
*wR*(*F* ^2^) = 0.108
*S* = 0.93
3000 reflections
175 parameters
H atoms treated by a mixture of independent and constrained refinement
Δρ_max_ = 0.39 e Å^−3^
Δρ_min_ = −0.37 e Å^−3^

### 

Data collection: *RAPID-AUTO* (Rigaku, 1998 ▶); cell refinement: *RAPID-AUTO*; data reduction: *RAPID-AUTO*; program(s) used to solve structure: *SHELXS97* (Sheldrick, 2008 ▶); program(s) used to refine structure: *SHELXL97* (Sheldrick, 2008 ▶); molecular graphics: *DIAMOND* (Brandenburg, 1999 ▶); software used to prepare material for publication: *SHELXL97*.

## Supplementary Material

Crystal structure: contains datablocks I, global. DOI: 10.1107/S1600536810015138/hy2300sup1.cif

Structure factors: contains datablocks I. DOI: 10.1107/S1600536810015138/hy2300Isup2.hkl

Additional supplementary materials:  crystallographic information; 3D view; checkCIF report

## Figures and Tables

**Table 1:** Hydrogen-bond geometry (Å, °)

*D*—H⋯*A*	*D*—H	H⋯*A*	*D*⋯*A*	*D*—H⋯*A*
N11—H10⋯Cl4^i^	0.86	2.60	3.433 (4)	164
N11—H11⋯Cl2^ii^	0.86	2.64	3.470 (4)	161
C3—H3⋯Cl4^iii^	0.99 (5)	2.89 (5)	3.864 (4)	169 (4)
C6—H6⋯Cl2^iv^	0.93	2.83	3.658 (4)	149

Symmetry codes: (i) 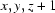; (ii) 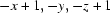; (iii) 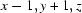; (iv) 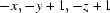.

**Fig. 1.**
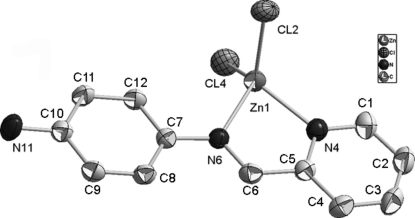
Molecular structure of the title compound. Displacement ellipsoids are drawn at the 50% probability level.

**Fig. 2.**
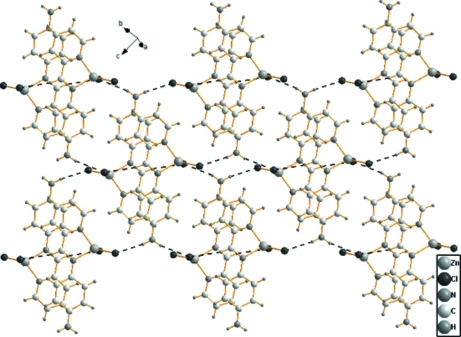
A view of the two-dimensional layer-like structure in the title compound.

## supplementary crystallographic information

### Comment

Based on the design and syntheses of zinc Schiff-base complexes and the potential applications of these materials as fluorescent probes (Su *et al.*, 1999; Ye *et al.*, 2005), the title compound has been obtained. As shown in Fig. 1, the asymmetric unit contains one Zn^II^ ion, one N-(pyridin-2-ylmethylene)benzene-1,4-diamine ligand and two Cl atoms. The Zn^II^ atom exhibits a distorted tetrahedral coordinate geometry formed by two N atoms from the ligand and two Cl atoms, with the Zn—N distances of 2.057 (3) and 2.070 (3)Å and the Zn—Cl distances of 2.2000 (13) and 2.2456 (12) Å. As shown in Fig. 2, the complex molecules are connected into a two-dimensional supramolecular layer-like structure via weak N—H···Cl and C—H···Cl hydrogen-bonding interactions (Table 1).

### Experimental

The ligand was prepared according to the previous method (Ye *et al.*, 2005). 1,4-Diaminobenzene (1.08 g, 10 mmol) was dissolved in methanol (20 ml), followed by addition of 2-pyridine carboxaldehyde (4.24 mg, 40 mmol). The mixture was stirred at room temperature for 2 h and filtered. The resulting yellow crystalline solid was washed with methanol several times and dried in air. A solution of ZnCl_2_.2H_2_O(14 mg, 0.08 mmol) in acetonitrile (5 ml) was allowed to diffuse slowly into a methylene chloride solution (10 ml) of the ligand (0.179 g, 0.625 mmol) in an H-shaped tube. Colorless crystals were obtained over a week.

### Refinement

H atoms bonded to C3, C4 and C11 were located from a difference Fourier map and refined isotropically. The other H atoms were positioned geometrically and refined as riding atoms, with C—H = 0.93 and N—H = 0.86 Å and with *U*_iso_(H) = 1.2*U*_eq_(C,N).

### Crystal data

**Table:**

[ZnCl_2_(C_12_H_11_N_3_)]	*Z* = 2
*M**_r_* = 333.53	*F*(000) = 336
Triclinic, *P*1	*D*_x_ = 1.671 Mg m^−^^3^
Hall symbol: -P 1	Mo *K*α radiation, λ = 0.71073 Å
*a* = 7.5004 (15) Å	Cell parameters from 2613 reflections
*b* = 9.1168 (18) Å	θ = 3.2–26.5°
*c* = 10.186 (2) Å	µ = 2.24 mm^−^^1^
α = 84.36 (3)°	*T* = 293 K
β = 82.27 (3)°	Block, red
γ = 74.19 (3)°	0.20 × 0.18 × 0.16 mm
*V* = 662.7 (3) Å^3^	

### Data collection

**Table:**

Rigaku R-AXIS RAPID diffractometer	3000 independent reflections
Radiation source: fine-focus sealed tube	2345 reflections with *I* > 2σ(*I*)
graphite	*R*_int_ = 0.027
ω scans	θ_max_ = 27.5°, θ_min_ = 3.2°
Absorption correction: multi-scan (*ABSCOR*; Higashi, 1995)	*h* = −9→9
*T*_min_ = 0.645, *T*_max_ = 0.699	*k* = −11→11
6557 measured reflections	*l* = −13→13

### Refinement

**Table:**

Refinement on *F*^2^	Primary atom site location: structure-invariant direct methods
Least-squares matrix: full	Secondary atom site location: difference Fourier map
*R*[*F*^2^ > 2σ(*F*^2^)] = 0.038	Hydrogen site location: inferred from neighbouring sites
*wR*(*F*^2^) = 0.108	H atoms treated by a mixture of independent and constrained refinement
*S* = 0.93	*w* = 1/[σ^2^(*F*_o_^2^) + (0.058*P*)^2^ + 0.9028*P*] where *P* = (*F*_o_^2^ + 2*F*_c_^2^)/3
3000 reflections	(Δ/σ)_max_ < 0.001
175 parameters	Δρ_max_ = 0.39 e Å^−^^3^
0 restraints	Δρ_min_ = −0.36 e Å^−^^3^

### Fractional atomic coordinates and isotropic or equivalent isotropic displacement parameters (Å^2^)

**Table:**

	*x*	*y*	*z*	*U*_iso_*/*U*_eq_	
Zn1	0.27968 (6)	0.38462 (4)	0.24431 (4)	0.03673 (14)	
Cl2	0.09004 (13)	0.22915 (11)	0.26242 (10)	0.0490 (2)	
Cl4	0.55281 (14)	0.29132 (12)	0.13201 (10)	0.0515 (3)	
N4	0.1317 (4)	0.6067 (3)	0.2044 (3)	0.0353 (6)	
N6	0.2715 (4)	0.4658 (3)	0.4284 (3)	0.0308 (6)	
C2	−0.0484 (6)	0.8248 (5)	0.0885 (4)	0.0492 (9)	
H2	−0.0941	0.8704	0.0098	0.059*	
C3	−0.0912 (6)	0.9047 (5)	0.2008 (4)	0.0508 (10)	
C4	−0.0201 (6)	0.8340 (4)	0.3174 (4)	0.0455 (9)	
C1	0.0631 (5)	0.6763 (4)	0.0925 (4)	0.0433 (8)	
H1	0.0912	0.6232	0.0157	0.052*	
C11	0.5458 (6)	0.1380 (4)	0.6210 (4)	0.0422 (8)	
N11	0.5896 (5)	0.0970 (4)	0.8533 (3)	0.0536 (9)	
H11	0.6555	0.0052	0.8408	0.064*	
H10	0.5698	0.1306	0.9314	0.064*	
C9	0.4073 (5)	0.3393 (4)	0.7684 (3)	0.0375 (7)	
H8	0.3895	0.3764	0.8524	0.045*	
C5	0.0910 (5)	0.6854 (4)	0.3156 (3)	0.0342 (7)	
C10	0.5160 (5)	0.1897 (4)	0.7488 (3)	0.0366 (7)	
C6	0.1730 (5)	0.6044 (4)	0.4344 (3)	0.0357 (7)	
H6	0.1536	0.6533	0.5131	0.043*	
C8	0.3268 (5)	0.4319 (4)	0.6659 (3)	0.0347 (7)	
H7	0.2548	0.5306	0.6812	0.042*	
C12	0.4640 (5)	0.2327 (4)	0.5178 (3)	0.0382 (8)	
H9	0.4845	0.1973	0.4330	0.046*	
C7	0.3523 (4)	0.3789 (4)	0.5390 (3)	0.0312 (7)	
H5	0.604 (6)	0.045 (5)	0.601 (4)	0.050 (12)*	
H4	−0.030 (7)	0.890 (6)	0.403 (5)	0.070 (14)*	
H3	−0.189 (7)	1.002 (6)	0.196 (5)	0.079 (16)*	

### Atomic displacement parameters (Å^2^)

**Table:**

	*U*^11^	*U*^22^	*U*^33^	*U*^12^	*U*^13^	*U*^23^
Zn1	0.0404 (2)	0.0338 (2)	0.0327 (2)	−0.00136 (17)	−0.00508 (16)	−0.00900 (15)
Cl2	0.0465 (5)	0.0419 (5)	0.0599 (6)	−0.0092 (4)	−0.0042 (4)	−0.0183 (4)
Cl4	0.0469 (5)	0.0540 (5)	0.0475 (5)	−0.0026 (4)	0.0052 (4)	−0.0188 (4)
N4	0.0341 (15)	0.0352 (15)	0.0347 (14)	−0.0062 (12)	−0.0034 (12)	−0.0027 (12)
N6	0.0322 (14)	0.0299 (13)	0.0291 (13)	−0.0059 (11)	−0.0029 (11)	−0.0030 (10)
C2	0.051 (2)	0.047 (2)	0.049 (2)	−0.0123 (18)	−0.0147 (18)	0.0131 (18)
C3	0.045 (2)	0.038 (2)	0.063 (3)	0.0000 (17)	−0.0106 (19)	0.0056 (18)
C4	0.049 (2)	0.0335 (18)	0.052 (2)	−0.0061 (16)	−0.0058 (18)	−0.0070 (16)
C1	0.042 (2)	0.048 (2)	0.0377 (18)	−0.0097 (16)	−0.0052 (15)	0.0034 (16)
C11	0.054 (2)	0.0295 (17)	0.0401 (19)	−0.0002 (16)	−0.0105 (16)	−0.0089 (15)
N11	0.077 (3)	0.0386 (17)	0.0373 (16)	0.0017 (16)	−0.0176 (16)	0.0005 (13)
C9	0.043 (2)	0.0383 (18)	0.0299 (16)	−0.0080 (15)	−0.0015 (14)	−0.0092 (14)
C5	0.0333 (17)	0.0323 (16)	0.0369 (17)	−0.0084 (14)	−0.0036 (14)	−0.0024 (13)
C10	0.0409 (19)	0.0344 (17)	0.0348 (17)	−0.0098 (15)	−0.0049 (14)	−0.0029 (14)
C6	0.0389 (19)	0.0352 (17)	0.0327 (16)	−0.0079 (14)	−0.0017 (14)	−0.0089 (13)
C8	0.0386 (18)	0.0311 (16)	0.0334 (16)	−0.0067 (14)	−0.0006 (14)	−0.0085 (13)
C12	0.045 (2)	0.0345 (17)	0.0333 (16)	−0.0035 (15)	−0.0057 (14)	−0.0109 (14)
C7	0.0309 (16)	0.0320 (16)	0.0314 (15)	−0.0084 (13)	−0.0054 (13)	−0.0026 (12)

### Geometric parameters (Å, °)

**Table:**

Zn1—N4	2.057 (3)	C11—C12	1.391 (5)
Zn1—N6	2.070 (3)	C11—C10	1.396 (5)
Zn1—Cl4	2.2000 (13)	C11—H5	0.87 (4)
Zn1—Cl2	2.2456 (12)	N11—C10	1.371 (4)
N4—C1	1.343 (4)	N11—H11	0.8600
N4—C5	1.356 (4)	N11—H10	0.8600
N6—C6	1.281 (4)	C9—C8	1.374 (5)
N6—C7	1.418 (4)	C9—C10	1.403 (5)
C2—C3	1.370 (6)	C9—H8	0.9300
C2—C1	1.384 (5)	C5—C6	1.472 (5)
C2—H2	0.9300	C6—H6	0.9300
C3—C4	1.394 (6)	C8—C7	1.395 (4)
C3—H3	0.99 (5)	C8—H7	0.9300
C4—C5	1.384 (5)	C12—C7	1.387 (5)
C4—H4	1.03 (5)	C12—H9	0.9300
C1—H1	0.9300		
			
N4—Zn1—N6	81.72 (11)	C10—C11—H5	125 (3)
N4—Zn1—Cl4	120.24 (9)	C10—N11—H11	120.0
N6—Zn1—Cl4	118.70 (9)	C10—N11—H10	120.0
N4—Zn1—Cl2	109.98 (9)	H11—N11—H10	120.0
N6—Zn1—Cl2	108.64 (8)	C8—C9—C10	121.2 (3)
Cl4—Zn1—Cl2	113.46 (4)	C8—C9—H8	119.4
C1—N4—C5	118.7 (3)	C10—C9—H8	119.4
C1—N4—Zn1	130.3 (3)	N4—C5—C4	122.1 (3)
C5—N4—Zn1	110.8 (2)	N4—C5—C6	116.2 (3)
C6—N6—C7	122.8 (3)	C4—C5—C6	121.7 (3)
C6—N6—Zn1	111.6 (2)	N11—C10—C11	121.2 (3)
C7—N6—Zn1	125.5 (2)	N11—C10—C9	120.4 (3)
C3—C2—C1	119.9 (4)	C11—C10—C9	118.4 (3)
C3—C2—H2	120.0	N6—C6—C5	119.3 (3)
C1—C2—H2	120.0	N6—C6—H6	120.3
C2—C3—C4	118.9 (4)	C5—C6—H6	120.3
C2—C3—H3	116 (3)	C9—C8—C7	120.4 (3)
C4—C3—H3	124 (3)	C9—C8—H7	119.8
C5—C4—C3	118.7 (4)	C7—C8—H7	119.8
C5—C4—H4	117 (3)	C7—C12—C11	121.2 (3)
C3—C4—H4	124 (3)	C7—C12—H9	119.4
N4—C1—C2	121.7 (4)	C11—C12—H9	119.4
N4—C1—H1	119.1	C12—C7—C8	118.8 (3)
C2—C1—H1	119.1	C12—C7—N6	117.2 (3)
C12—C11—C10	120.0 (3)	C8—C7—N6	124.0 (3)
C12—C11—H5	115 (3)		
			
N6—Zn1—N4—C1	179.7 (3)	C3—C4—C5—N4	−0.4 (6)
Cl4—Zn1—N4—C1	61.3 (3)	C3—C4—C5—C6	179.1 (3)
Cl2—Zn1—N4—C1	−73.4 (3)	C12—C11—C10—N11	177.7 (4)
N6—Zn1—N4—C5	−5.4 (2)	C12—C11—C10—C9	−1.9 (6)
Cl4—Zn1—N4—C5	−123.9 (2)	C8—C9—C10—N11	−177.6 (3)
Cl2—Zn1—N4—C5	101.5 (2)	C8—C9—C10—C11	2.0 (5)
N4—Zn1—N6—C6	4.4 (2)	C7—N6—C6—C5	−178.8 (3)
Cl4—Zn1—N6—C6	124.4 (2)	Zn1—N6—C6—C5	−2.7 (4)
Cl2—Zn1—N6—C6	−104.0 (2)	N4—C5—C6—N6	−2.0 (5)
N4—Zn1—N6—C7	−179.7 (3)	C4—C5—C6—N6	178.4 (3)
Cl4—Zn1—N6—C7	−59.6 (3)	C10—C9—C8—C7	−0.3 (5)
Cl2—Zn1—N6—C7	72.0 (3)	C10—C11—C12—C7	0.1 (6)
C1—C2—C3—C4	0.3 (6)	C11—C12—C7—C8	1.7 (5)
C2—C3—C4—C5	−0.1 (6)	C11—C12—C7—N6	−178.6 (3)
C5—N4—C1—C2	−0.5 (5)	C9—C8—C7—C12	−1.6 (5)
Zn1—N4—C1—C2	174.1 (3)	C9—C8—C7—N6	178.8 (3)
C3—C2—C1—N4	0.0 (6)	C6—N6—C7—C12	−176.7 (3)
C1—N4—C5—C4	0.7 (5)	Zn1—N6—C7—C12	7.8 (4)
Zn1—N4—C5—C4	−174.9 (3)	C6—N6—C7—C8	3.0 (5)
C1—N4—C5—C6	−178.8 (3)	Zn1—N6—C7—C8	−172.5 (2)
Zn1—N4—C5—C6	5.6 (3)		

### Hydrogen-bond geometry (Å, °)

**Table:**

*D*—H···*A*	*D*—H	H···*A*	*D*···*A*	*D*—H···*A*
N11—H10···Cl4^i^	0.86	2.60	3.433 (4)	164
N11—H11···Cl2^ii^	0.86	2.64	3.470 (4)	161
C3—H3···Cl4^iii^	0.99 (5)	2.89 (5)	3.864 (4)	169 (4)
C6—H6···Cl2^iv^	0.93	2.83	3.658 (4)	149

Symmetry codes: (i) *x*, *y*, *z*+1; (ii) −*x*+1, −*y*, −*z*+1; (iii) *x*−1, *y*+1, *z*; (iv) −*x*, −*y*+1, −*z*+1.
